# Increased aggression and reduced aversive learning in honey bees exposed to extremely low frequency electromagnetic fields

**DOI:** 10.1371/journal.pone.0223614

**Published:** 2019-10-10

**Authors:** Sebastian Shepherd, Georgina Hollands, Victoria C. Godley, Suleiman M. Sharkh, Chris W. Jackson, Philip L. Newland

**Affiliations:** 1 Biological Sciences, University of Southampton, Highfield Campus, Southampton, United Kingdom; 2 Department of Entomology, Purdue University, West Lafayette, Indiana, United States of America; 3 Mechatronics, Mechanical Engineering, University of Southampton, Highfield Campus, Southampton, United Kingdom; University of Illinois at Urbana-Champaign, UNITED STATES

## Abstract

Honey bees, *Apis mellifera*, are a globally significant pollinator species and are currently in decline, with losses attributed to an array of interacting environmental stressors. Extremely low frequency electromagnetic fields (ELF EMFs) are a lesser-known abiotic environmental factor that are emitted from a variety of anthropogenic sources, including power lines, and have recently been shown to have a significant impact on the cognitive abilities and behaviour of honey bees. Here we have investigated the effects of field-realistic levels of ELF EMFs on aversive learning and aggression levels, which are critical factors for bees to maintain colony strength. Bees were exposed for 17 h to 100 μT or 1000 μT ELF EMFs, or a sham control. A sting extension response (SER) assay was conducted to determine the effects of ELF EMFs on aversive learning, while an intruder assay was conducted to determine the effects of ELF EMFs on aggression levels. Exposure to both 100 μT and 1000 μT ELF EMF reduced aversive learning performance by over 20%. Exposure to 100 μT ELF EMFs also increased aggression scores by 60%, in response to intruder bees from foreign hives. These results indicate that short-term exposure to ELF EMFs, at levels that could be encountered in bee hives placed under power lines, reduced aversive learning and increased aggression levels. These behavioural changes could have wider ecological implications in terms of the ability of bees to interact with, and respond appropriately to, threats and negative environmental stimuli.

## Introduction

Over the last 30 years there has been a decline in the numbers of the economically and ecologically important honey bee [1, 2]. Honey bee declines are part of a much larger global problem of pollinator declines [3] with major causes attributed to a combination of interacting, and mainly anthropogenically driven, environmental stressors including, habitat loss, pesticide exposure, pathogens and parasites [4]. Electromagnetic pollution is emerging as a lesser-known abiotic environmental factor that has the potential to affect insect biology and thus may contribute to the environmental stress load that insects currently experience in global ecosystems [5, 6].

Extremely low frequency electromagnetic fields (ELF EMFs) are a specific type of non-ionising electromagnetic radiation in the frequency range 3–300 Hz that are emitted from anthropogenic devices. Pollution of the environment with ELF EMFs has increased dramatically in the last century, with a major source for ELF EMFs being power transmission lines [7]. ELF EMF exposure has recently been associated with a variety of different effects on insects including changes in developmental biology [8, 9], locomotor behaviour [6, 10], molecular biology [11, 12], and immune response [13].

Honey bees may be particularly at risk to ELF EMF pollution in the environment. At ground level, ELF EMF intensity under power transmission lines can reach 100 μT, while flying insects can be exposed to much higher levels close to conductors where ELF EMF levels can be over 1,000 μT [5]. Some studies suggest exposure to ELF EMFs from power lines may be stressful for honey bees [14, 15] whilst it has also been reported [16] that bees hived under power lines will readily abscond. Moreover, Greenberg et al. [17] found that bee hives exposed to power lines had increased motor activity, abnormal propolisation, reduced weight gain of hives, queen loss, impaired production of queen cells, decreased sealed brood and poor winter survival, leading to a federal US precaution to not store hives under power lines [18]. While these studies show no direct experimental evidence for ELF EMF effects on bees, they at least suggest that ELF EMF exposure may be a factor that contributed to, or caused, the stress responses of the bees observed in these studies.

In their environment bees are exposed to a variety of negative environmental stimuli and cues, which are also critical for bees to perceive and respond to, such as weather, toxins [19], or biotic threats such as colony diseases and parasites [20, 21], invading robber bees from other colonies [20] and predators [21–23]. How colonies respond to these environmental stresses is critical to their long-term fitness. Bees must be able to detect these negative stimuli [20], learn that they are associated with a negative effect [19], enact an appropriate aggressive response [22], and even communicate this information to other individuals [23]. For example, guard bees when confronted with a threat (e.g. predator or intruder) may enter the hive to release alarm pheromone by extruding their sting, raising their abdomen and fanning their wings [24, 25].

Surprisingly little is known about aversive learning, and how it is affected by environmental stimuli, despite its importance in maintaining colony fitness. A sting extension response (SER) assay [26, 27] has been developed to study aversive learning in bees in which a conditioned stimulus (CS) (often olfactory) is applied and associated with an unconditioned stimulus (US) of a weak electric shock. Over repeated conditioning trials bees learn to associate the negative US with the CS. The SER assay can therefore provide valuable information in a controlled experimental environment of how potential stressors such as ELF EMFs can affect bees [28]. For example, SER has been used to investigate the impacts of the neonicotinoid insecticide imidacloprid on honey bee aversive learning [29]. In addition, intruder assays have been used to assess aggressive responses of honey bees, including to conspecifics [30–33]. Environmental stresses which could affect the ability of bees to learn about negative environmental cues, or respond appropriately to environmental cues, could therefore be detrimental to honey bee colony health.

Here we have used both the SER and intruder assays to determine whether short term exposure to ELF EMFs, at levels equivalent to those found at ground level under high-voltage transmission power lines, can affect aversive learning and aggression in honey bees. We have utilised these well-established assays in the laboratory where the levels of EMF exposure of individual bees can be precisely controlled, and under consistent conditions free from stray fields and other confounding stimuli.

## Materials and methods

### Magnetic fields

Electromagnetic fields were generated with a custom-made Helmholtz coil [5] which produced homogenous 50 Hz sinusoidal AC electromagnetic fields with a range of field strength from ~10 μT—10,000 μT. Field strength (magnetic flux density) was measured with a Model GM2 Magnetometer (Alphalab Inc., USA). For control exposures no current was passed through the coil system. For SER experiments control, 100 μT and 1000 μT 50 Hz EMF treatments were applied, while for intruder assay experiments control and 100 μT ELF EMF treatments were used.

### Animals

Honey bees were kept at the University of Southampton Highfield Campus apiary (50° 56' 10''N, 1° 23' 39''W) and experiments conducted from June-August, 2017. Foragers were identified by the pollen in their corbiculae and transported to an insectary in the Institute for Life Sciences at the University of Southampton, where they were immobilized on wet ice and transferred into appropriate containers for SER and Intruder Assay experiments.

### Sting extension response assay

**Fig 1:**
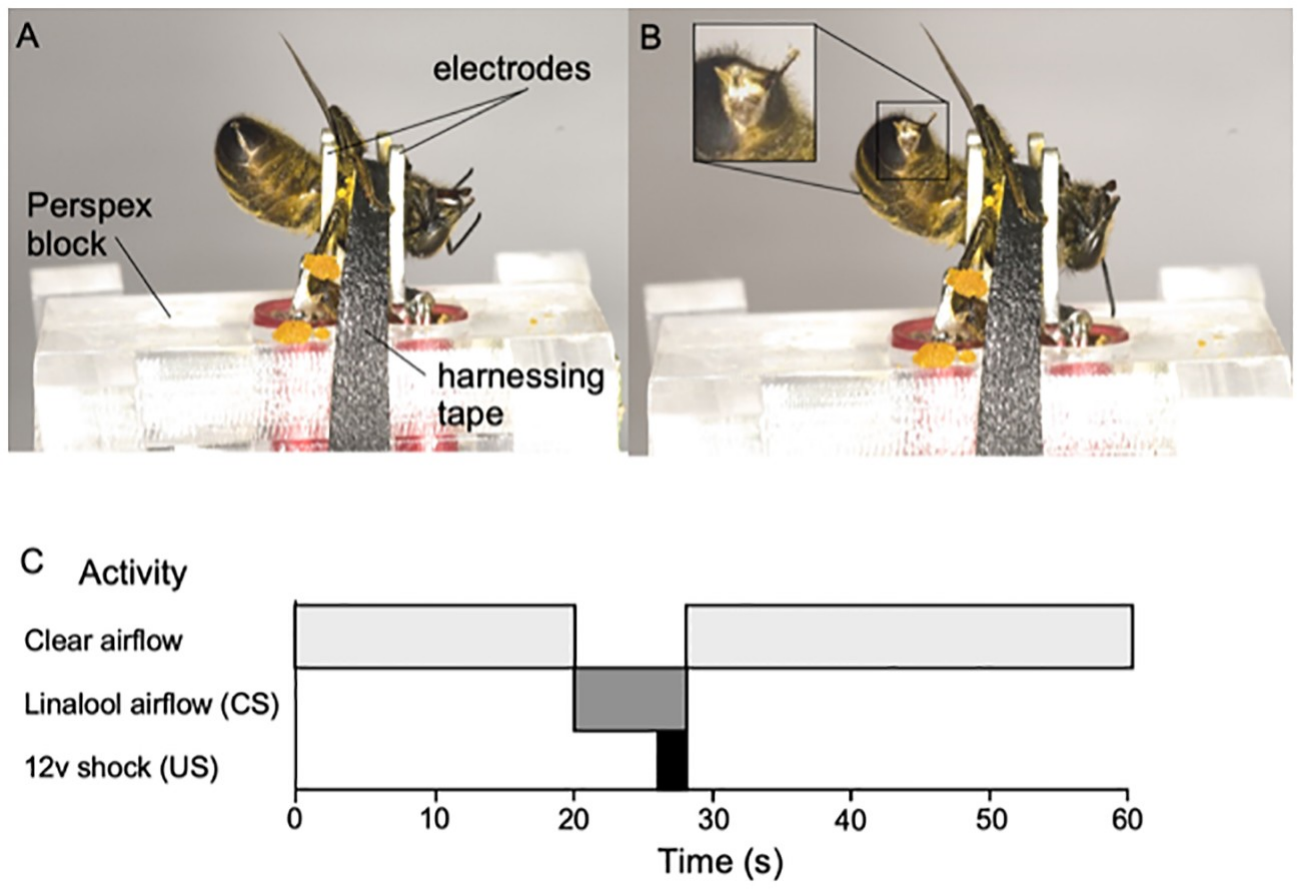
Sting extension response protocol. A) Harnessing of a bee in an SER cradle for EMF exposure. Tesa^©^ tape was applied around the thorax to hold the bee between the fork prongs. B) Aversive sting extension response to the CS in SER conditioning trials. The inset shows the extended stinger in more detail. C) SER Timetable showing a representation of an individual conditioning trial. The bee was acclimatised to the arena for 20 s, before CS (linalool) application. After 6 s of CS, CS and US (12 V shock) were paired for 2 s, after which both CS and US were switched off. A further 32 s of clear airflow was allowed for odour to be removed from the arena.

Bees were collected individually from 3 hives and harnessed in custom made SER cradles cut from Perspex, with a similar design to Vergoz et al. [27]. Bees were placed ventral side upwards in a metal fork of the cradle, such that the fork held the bee by the thorax, with prongs in place around the petiole and neck of the bee (Fig 1A). This fork also served as an electrode for an aversive shock stimulus during the SER assay (Fig 1B). Tesa^©^ tape was then placed laterally across the cradle and between the prongs of the fork across the thorax to restrain the bee in the cradle. Bees were then fed to satiation with a 50% w/v sucrose solution and were then ready for overnight treatment (17 h).

An experimental arena (W × D × H = 60 × 45 × 55 cm) was used with an odour delivery system at one end and an extraction fan at the other to remove any odours from the arena. The odour delivery system allowed a constant airflow to be supplied to the arena. A clear airflow, and the CS, were delivered in separate channels in the multichannel system which joined via Teflon tubing before it discharged into the arena at a single release point. Electronic valves allowed the airflow to switch between CS and clear airflow channels. The CS used was 8 μl of 97% linalool (Sigma-Aldrich, UK) which was pipetted onto filter paper to be placed in the CS delivery channel. The channel with clear air was always open when no odour was delivered. To deliver the CS, airflow was switched from the clear air channel to the odour delivery channel such that bees were supplied with a constant airflow, and would associate any stimulus with the odour and not a change in airflow.

For SER experiments bees were exposed to control, 100 μT or 1000 μT EMFs for 17 h and following exposure SER trials began immediately. This treatment was chosen to represent a field-realistic scenario where bee hives are placed under transmission and where bees have been reported to show negative responses [17]. 357 bees completed the SER assay. An SER cradle containing a harnessed bee was placed into the experimental arena of the odour delivery system. Bees were exposed to a clear airflow for 20 s (Fig 1C). During this time the SER cradle was attached to a DC power-supply with a 12 V output. The airflow was then switched from clean air to linalool airflow, representing the CS. The CS lasted 8 s. For the final 2 s of the CS the bee was shocked at 12 V from the DC power supply, representing the unconditioned stimulus (US) thus pairing US and CS for 2 s. The US and CS finished at the same time (28 s into the trial). The clear airflow was then left on for 32 s with the bee in the arena to reinforce the association of the CS with the US and to allow the extractor to remove linalool from the arena. The length of one complete conditioning trial for a bee was 60 s (Fig 1C).

Conditioning trials were repeated 5 times for each individual bee with an inter-trial interval of 10 min. If a bee did not respond during linalool delivery or electric shock then a ‘failed response’ was recorded. Bees that failed to respond more than once in conditioning trials (n = 16, 4.5% of 357) were excluded from analyses. No bees exhibited a pre-learned aversive response to linalool in the first conditioning trial, and therefore no bees had to be excluded from analysis for this reason. After all exclusions were made, 341 bees remained that completed the SER assay for inclusion in statistical analyses (S1 Table).

If a bee responded only after the shock stimulus then a non-conditioned sting extension response was recorded (i.e. the bee responded to US but not CS). As in previous aversive learning studies responses to the conditioned stimulus have been described only when a bee extends its sting during the CS application, and are defined as a ‘sting extension response’ (Fig 1A and 1B). The proportions of conditioned sting extension responses over 5 trials were analysed to assess the effects of short-term ELF EMF exposure on aversive learning in honey bees.

This aversive learning approach therefore measures acquisition and short-term retention of information, and thus has comparability with the results of the intruder assay where bees encounter a new individual from a foreign hive.

### Intruder assay

Bees were collected from 5 different hives in groups of 20 bees from the same hive of origin. Each group of 20 was split into 2 paired cohorts of 10 (S2 Table), and stored in separate petri dishes fitted with 50% w/v sucrose feeders. For each pair of 10-bee cohorts (from the same hive of origin) 1 cohort was exposed to a 100 μT ELF EMF and the other exposed to control conditions (both at 22 ± 1°C) for 17 h overnight. The intruder assay was conducted the next day.

The sample period for the intruder assay began when a forager bee from a 6^th^ (and different) hive was introduced into each petri dish. Focal sampling of the ‘intruder’ bee was conducted continuously for 10 min to assess the behaviour of recipient bees towards the intruder. Behaviours were categorized on an aggressive severity index adapted from Richard et al. [31] (Table 1) and the aggressive severity indices summed for a full 10 min sample period to give an overall aggression score for that sample. In total 60 intruder assay samples were conducted (n = 30 per treatment, with 6 assays/treatment/hive).

**Table 1:** Aggressive severity behavioural index used in the intruder assay adapted from Richard et al. [31].

Behaviour	Definition	Aggressive Severity Index
Aggressive antennation	Antennation directed towards the intruder or touching the intruder with antennae	1
Stalking	Follows and moves towards intruder for more than 5 seconds	1
Crawl over	Moves directly on top of the intruder	1
Antennation with mandibles open	Antennation directly towards the intruder with mandibles open	2
Biting	Uses mandibles to grasp the intruder	3
Abdomen flexion	The abdomen is flexed but the stinger is not extruded	4
Stinging attempts	The stinger is visibly extruded towards the intruder	5

### Statistical analysis

Data were analysed in SPSS (v.24, IBM SPSS Inc.) and Graphpad Prism (v.7, Graph Pad Software Inc.). Where appropriate, homogeneity of variance and normality assumptions were tested. For all models assessing the effects of treatments on binomial SER data, binomial error structure and logit link function were used, and where appropriate pairwise contrasts with Bonferroni adjusted significance were used in *post-hoc* analyses.

To determine whether ELF EMF exposure or ‘hive or origin’ affected the initial aversive responsiveness of bees a generalized linear model (GLM) with ‘EMF treatment’ and ‘hive of origin’ as interacting factors was used. To analyse the effect of ELF EMF exposure on sting extension responses, a generalized mixed effect model (GLMM) was used with ‘EMF treatment’, ‘hive of origin’, and ‘conditioning trial’ as interacting factors. For GLMMs trial 1 was not included in analyses (i.e. trials 2–5 were used), as learning cannot occur in the first trial. For intruder assay analysis, aggression scores were totalled from each trial and data log_10_-transformed to satisfy normality assumptions for parametric statistical analyses. A two-way Repeated Measures ANOVA was conducted to determine the effects of ‘EMF’, and ‘Hive of Origin’ on log-transformed aggression score data, with data paired by their collection cohort. Data plotted in aggression score graphs is back-transformed.

## Results

### Sting extension response

#### ELF EMFs do not reduce the ability of bees to respond to aversive stimuli

**Fig 2:**
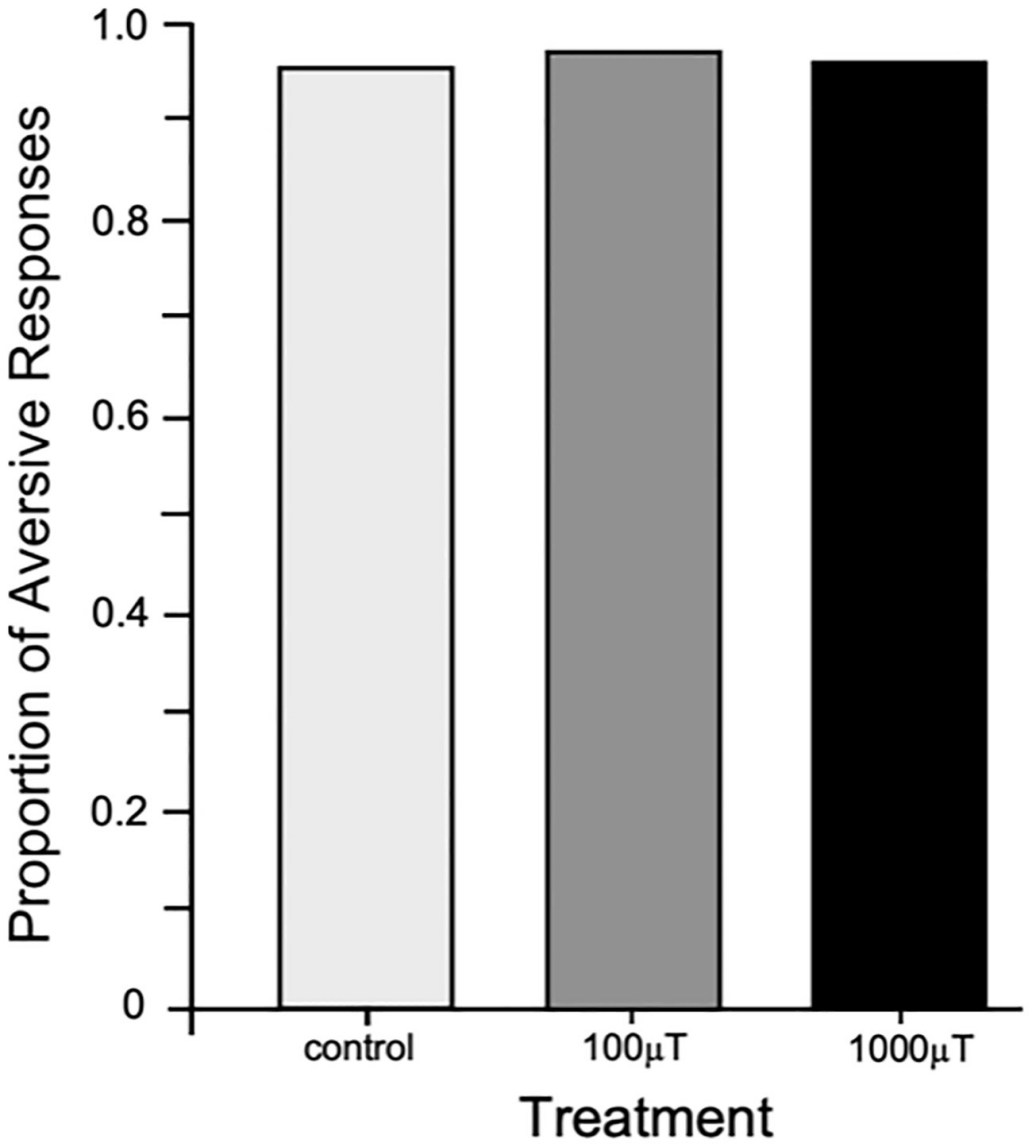
Aversive responses of honey bees in the SER assay. The effect of ELF EMF treatment on the proportion of aversive responsiveness to 12 V electric shock aversive stimuli. Exact proportions are plotted. Results show that ELF EMFs had no effect on the aversive responses of bees to electrical stimulation.

To determine whether short-term exposure to EMF (control, 100 μT, or 1000 μT) affected the ability of bees to respond with an aversive extension of the sting, the proportions of bees which did not fail to respond to the US (i.e. non-learned sting extension to an aversive stimulus) between each treatment were compared. After 17 h control exposure 95.0% of bees (n = 119) exhibited aversive responses (Fig 2), whereas 96.6% (n = 118) responded following exposure to 100 μT and 95.0% (n = 120) responded following exposure to 1000 μT EMFs. Thus, the initial aversive responsiveness of honey bees was not affected by any interaction between the ELF EMF ‘treatment’ or the honey bee ‘hive of origin’ (GLM, χ^2^<0.001, d.f. = 4, P > 0.99), nor were there any main effects of ‘treatment’ (GLM, χ^2^<0.001, d.f. = 2, P > 0.99) or ‘hive of origin’ (GLM, χ^2^<0.001, d.f. = 2, P > 0.99).

#### ELF EMFs reduce learning performance of the sting extension response

**Fig 3:**
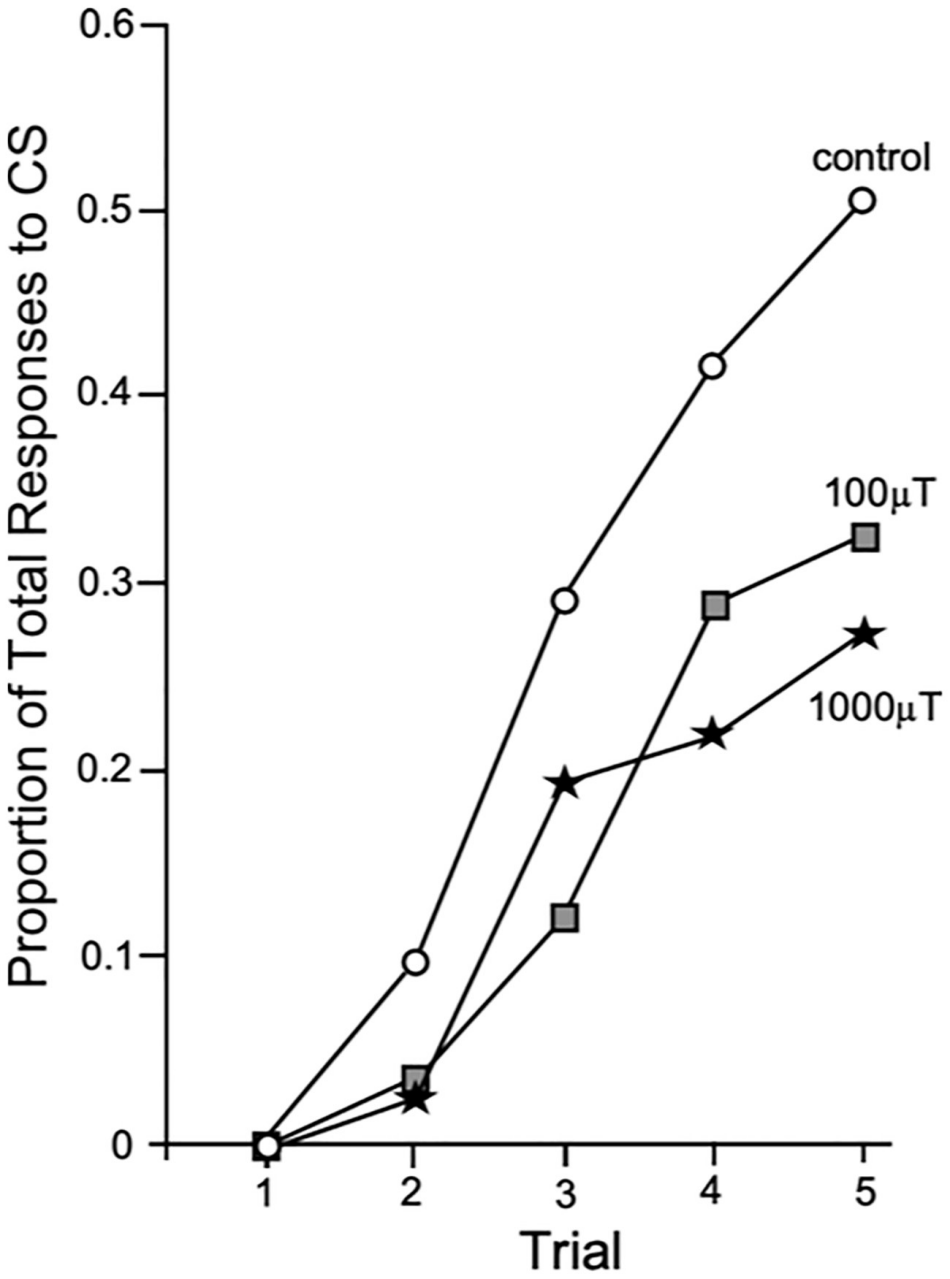
Effects of ELF EMFs on aversive learning in honey bees. Effect of short-term ELF EMF exposure on the proportion of aversive responses to the conditioned stimulus (linalool) for each of the trials. For each treatment the proportion of bees showing a learned response increased. The exact proportion of responses is plotted.

For control bees, and those exposed to 100 μT and 1000 μT ELF EMFs, the proportion of bees exhibiting a sting extension response increased with each conditioning trial (GLMM, F_3,1352_ = 26.08, P < 0.0001). For bees maintained under control conditions 29% showed SER after trial 3 while 50% showed SER after conditioning trial 5 (Fig 3). By contrast, after bees were exposed to 100 μT ELF EMFs only 12% of bees showed SER after trial 3 and 32% after trial 5. Following exposure to 1000 μT ELF EMFs 19% showed an SER after trial 3 and 27% after trial 5. EMF treatments were found to significantly reduce the proportions of SER in honey bees (GLMM, F_2,1352_ = 15.01, P < 0.0001). A greater proportion of control exposed bees exhibited SER than both 1000 μT (Pairwise comparison, Bonferroni adjusted P < 0.001) and 100 μT (Pairwise comparison, Bonferroni adjusted P = 0.001) exposed bees. There was no ‘treatment’ * ‘trial’ interaction (GLMM, F_1,1352_ = 0.82, P = 0.56).

In this analysis of the effects of ELF EMF exposure on sting extension responses, hive of origin was removed as a factor to improve model fit as it was found to have no effect on the proportion of SER to the CS (GLMM, F_2,1328_ = 0.17, P = 0.84), nor any interaction with ‘treatment’ (GLMM, F_4,1328_ = 1.38, P = 0.24) ‘conditioning trial’ (GLMM, F_6,1328_ = 0.24, P = 0.96) or three-way interaction (GLMM, F_12,1328_ = 0.33, P = 0.99).

### Intruder assay

**Fig 4:**
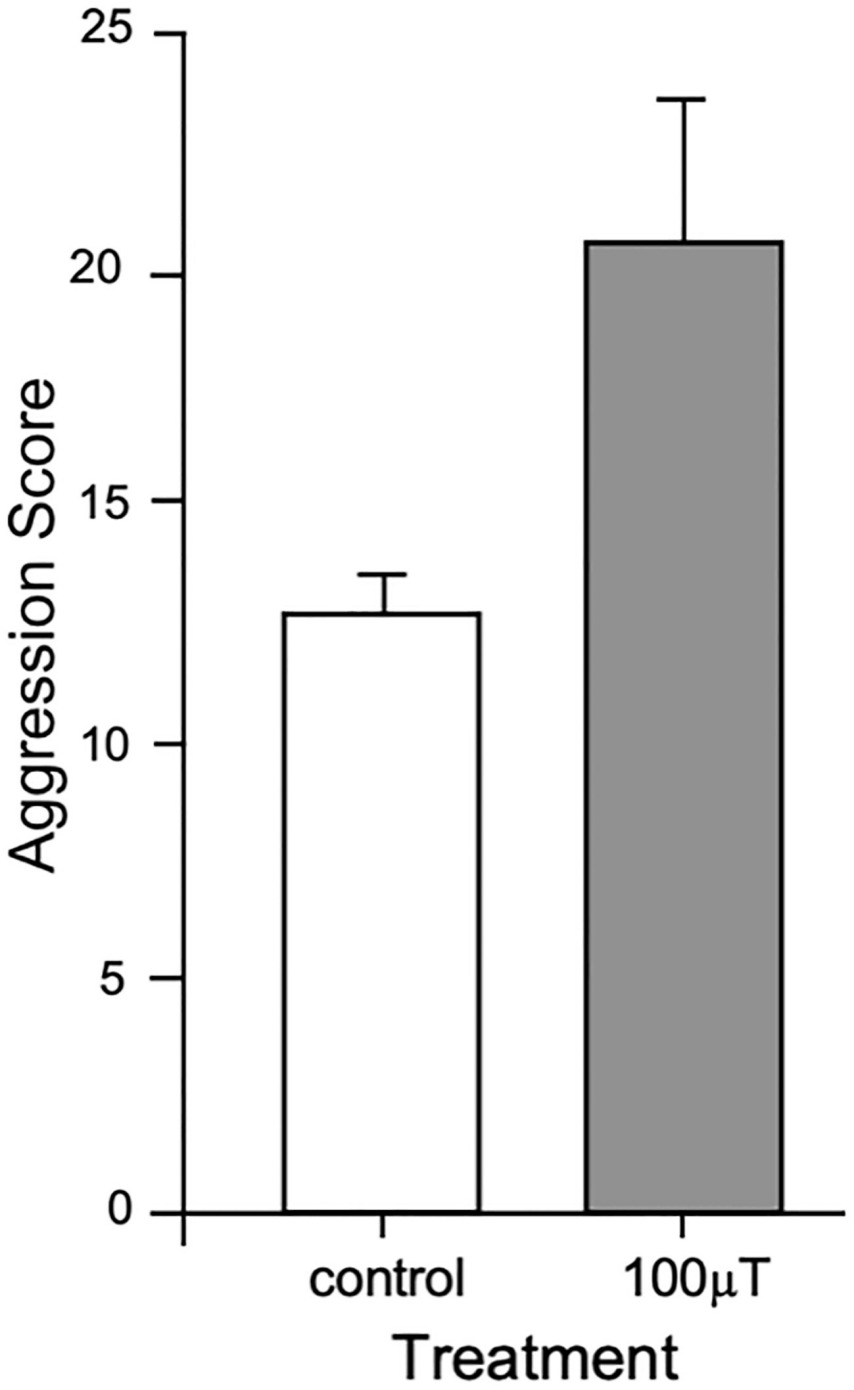
The effect of ELF EMFs on honey bee aggression levels. Exposure to a 100 μT ELF EMF significantly increased the Aggression Score. Mean ± SEM are shown. Statistical analyses were conducted on log-transformed data. Data plotted are reverse log-transformed from data used in statistical analysis.

Bees exposed to 100 μT ELF EMF exhibited greater aggressive behaviour to introduced bees, than bees not exposed to ELF EMFs (Fig 4). Bee cohorts which received a control treatment displayed an aggression score of 12.87 ± 1.69 (mean ± SEM) whereas bee cohorts exposed to 100 μT EMF exhibited a mean aggression score of 20.70 ± 2.14 (mean ± SEM, Standard Error of the Mean). EMF exposure significantly increased the average aggression scores across bees from all hives (F_1,25_ = 11.42, P = 0.0024). There was no impact of *Hive* (F_4,25_ = 0.65, P = 0.63) or any Hive*EMF interaction effect (F_4,25_ = 0.75, P = 0.56) on aggression score. This indicates that short-term ELF EMF exposure, at levels that can be encountered at ground level or in proximity to a high voltage transmission power lines, led to an increase in aggressive behaviour of bees directed towards conspecifics.

## Discussion

Short-term exposure to 50 Hz ELF EMFs reduced aversive learning performance and increased aggression at levels as low as 100 μT. This directly shows, for the first time, that short-term ELF EMF exposure at levels which can be encountered at ground level under high-voltage transmission power lines can affect honey bees, in terms of both their conditioning to negative stimuli, and the intensity of their aggressive behaviour.

In locusts ELF EMFs have been shown to affect neural circuits controlling limb movement and muscular force [6]. During the stinging response in honey bees the protraction of the tip of the abdomen, and the alternate sliding of barbed lancets of the stinging apparatus, are coordinated by four large abdominal muscles [34–36] whose activity are regulated by neural circuits in the terminal abdominal ganglion [22]. Given that a sting extension response was evoked by the US in over 95% of trials, it is unlikely that the effects on aversive learning were due to the effects of EMF at the neuromuscular level. Similarly, the effects of EMF were not due to changes in the sting extension motor pattern as bees could still extend their abdomens to electric shocks. Instead ELF-EMF induced reductions in SER performance are solely down to a reduced ability to learn the aversive stimuli, and not the motor pattern involved in responding to the stimuli.

The mechanisms underlying the effects of ELF EMFs on honey bee aversive learning and aggression may be diverse. While the neural pathways underlying appetitive learning in the honey bee brain are well characterised [37, 38], less is known of the neural architecture underlying aversive learning. The biogenic amines dopamine and octopamine have critical roles in associative learning in honey bees [39]. Vergoz et al. [27] for example, found that aversive learning is impaired after the injection of dopaminergic antagonists, and Jarriault et al. [40] found that dopamine was released in mushroom bodies in the honey bee brain after electric shock stimulation of the abdomen. These findings suggest that dopamine may have a key role in memory formation in honey bee aversive learning. Furthermore, the honey bee alarm pheromone has been shown to increase levels of the biogenic amines serotonin and dopamine, with increases in these amine levels being associated with increased likelihood of a bee to sting [41]. Some studies investigating the effects of EMF on invertebrates have suggested that increased biogenic amine levels lead to increases in behavioural activity [42, 43]. While no studies have yet analysed changes in dopamine levels following ELF EMF exposure, these previous studies suggest that biogenic amine levels may be a potential area to investigate to elucidate the underlying mechanisms of ELF EMF induced changes in insect behaviour. Moreover, ELF EMFs have been shown to have effects on neuronal signalling in insects [6], and therefore there is the potential for ELF EMF induced effects on dopaminergic neurons or other neural circuits which are involved in aversive learning pathways. ELF EMF induced changes in behaviour could also be underpinned by molecular changes such as gene expression. For example short-term ELF EMF exposure has been shown to increase heat-shock protein expression in locusts [6] and *Drosophila* [12].

The ecological implications of these effects are diverse. On the one hand the reduced ability to learn new negative stimuli could lead to an increased latency of honey bee colonies to respond to novel threats. Maliszewska et al. [10] found that short-term exposure of American cockroaches to 7,000 μT ELF EMFs increased the latency of responses to a negative heat stimulus. The increase in latency could clearly be detrimental to individuals in the ability to avoid harmful environmental stimuli. On the other hand, we found that bees exposed to ELF EMFs showed increased aggression levels. Rittschof et al. [33] found that increased levels of aggression in honey bees are associated with greater resilience to environmental stresses and to immune challenge. However, direct short-term ELF EMF exposure at 2,000 μT in Lepidopteran larvae has been associated with changes in immune response parameters such as increased apoptotic-like hemocytes, reduced hemolyph total protein and reduced hemocyte cell count, which could suggest short-term ELF EMF exposures might lead to reduced resilience to immune challenge [13]. It is not known if ELF EMFs affect immune response in honey bees at field-realistic ELF EMF intensities, lower than those that have been studied with Lepidoptera, and thus it is not known if ELF EMF exposure would confer greater resilience to immune challenge alongside increased aggression levels in bees. In addition, in the environment if a bee perceives a negative stimulus a sting response often results in sting autonomy, with a rupture of the abdomen that causes the eventual death of the bee [44, 45]. Less aggressive responses to negative stimuli such as aggressive buzzing and flight bombardment can be successful methods of warding off threats in a manner that is less detrimental to a colony in terms of bee loss [25, 45]. The effects of environmental stressors and the consequences of increased aggression on this aversive decision making processes (other than increased sting autonomy) are not-known.

While it is unclear what the ecological consequences of increased aggression may be for bees exposed to ELF EMFs, the implications of reduced aversive learning performance are more distinct. It is imperative that honeybees are able to perceive, learn, and avoid threats in the environment [28, 39]. Reductions in the ability to learn about negative stimuli could have implications for the abilities of bees to deal with predatory/invader threats [20, 22], detecting/avoiding deleterious stimuli [19] and responding to negative stimuli that require action e.g. attacking/removing diseased individuals from the hive [20], all of which could have detrimental effects on bee colonies. Although it is not yet known how bees will actually respond in the field, it is clear that the reduction in aversive learning seen here with short-term 100 μT exposures could be detrimental to honeybees on an ecological level. A number of studies have described bee colonies failing that are hived under high-voltage transmission power lines, where EMF levels can reach 100 μT [14–17]. There is the possibility that with hives located under power lines, the long-term chronic exposure to ELF EMFs could continually reduce cognitive abilities both with regards to aversive and appetitive learning, potentially leading to some of the negative effects found in these studies.

Reductions in learning could be detrimental to individual and colony survivability. There are large potential ecological consequences for reduced ability to learn about aversive and appetitive stimuli for bees. Future studies should focus on whether there are ecological effects of ELF EMF exposure, with direct measurements of chronic EMF exposure under power lines, as well as determining what physiological/molecular processes may be affected by this kind of exposure. These effects may not be confined to managed honey bees as there may be much wider implications for wild bees and even other pollinators that require power line strips for critical habitat refuge [46–50]. The underlying mechanisms, as well as the potential ecological implications of ELF EMF pollution in the field must be further investigated to determine the effects of ELF EMF pollution on insect biology and ecology, including crucial pollination ecosystem services.

## Supporting information

S1 Table The number of bees in SER analyses (after exclusions) for each hive and treatment. (DOCX)

S2 Table The number of bees in intruder assay analyses for each hive and treatment. (DOCX)

S1 Dataset Datasets for A) SER data B) Aggression data. (XLSX)

## References

[1] Hayes J, Underwood RM, Pettis J. A survey of honey bee colony losses in the US, fall 2007 to spring 2008. PLoS One, 2008;3(12): e4071 10.1371/journal.pone.0004071 19115015 PMC2606032

[2] Potts SG, Roberts SP, Dean R, Marris G, Brown MA, Jones R, et al Declines of managed honey bees and beekeepers in Europe. J Apic Res. 2010;49(1): 15–22.

[3] Hallmann CA, Sorg M, Jongejans E, Siepel H, Hofland N, Schwan H, et al More than 75 percent decline over 27 years in total flying insect biomass in protected areas. PLoS One. 2017;12(10): e0185809 10.1371/journal.pone.0185809 29045418 PMC5646769

[4] Goulson D, Nicholls E, Botías C, Rotheray EL. Bee declines driven by combined stress from parasites, pesticides, and lack of flowers. Science. 2015;347(6229): 1255957 10.1126/science.1255957 25721506

[5] Shepherd S, Lima MA, Oliveira EE, Sharkh SM, Jackson CW, Newland PL. Extremely low frequency electromagnetic fields impair the cognitive and motor abilities of honey bees. Sci Rep. 2018;8(1): 7932 10.1038/s41598-018-26185-y 29785039 PMC5962564

[6] Wyszkowska J, Shepherd S, Sharkh S, Jackson CW, Newland PL. Exposure to extremely low frequency electromagnetic fields alters the behaviour, physiology and stress protein levels of desert locusts. Sci Rep. 2016;6: 36413 10.1038/srep36413 27808167 PMC5093409

[7] World Health Organization. Extremely low frequency fields—Environmental Health Criteria. Geneva: World Health Organization Press; 2007.

[8] Dimitrijević D, Savić T, Anđelković M, Prolić Z, Janać B. Extremely low frequency magnetic field (50 Hz, 0.5 mT) modifies fitness components and locomotor activity of *Drosophila subobscura*. Int J Radiat Biol. 2014;90(5): 337–43. 10.3109/09553002.2014.888105 24475738

[9] Zmejkoski D, Petković B, Pavković-Lučić S, Prolić Z, Anđelković M, Savić T, 2017 Different responses of *Drosophila subobscura* isofemale lines to extremely low frequency magnetic field (50 Hz, 0.5 mT): fitness components and locomotor activity. Int J Radiat Biol. 2017;93(5): 544–52. 10.1080/09553002.2017.1268281 27921519

[10] Maliszewska J, Marciniak P, Kletkiewicz H, Wyszkowska J, Nowakowska A, Rogalska J. Electromagnetic field exposure (50 Hz) impairs response to noxious heat in American cockroach. J Comp Physiol A. 2018;204(6): 605–11. 10.1007/s00359-018-1264-2 PMC5966488 29721708

[11] Todorović D, Mirčić D, Ilijin L, Mrdaković M, Vlahović M, Prolić Z, et al Effect of magnetic fields on antioxidative defense and fitness-related traits of *Baculum extradentatum* (insecta, phasmatodea). Bioelectromagnetics. 2012;33(3): 265–73. 10.1002/bem.20709 21953292

[12] Li SS, Zhang ZY, Yang CJ, Lian HY, Cai P. Gene expression and reproductive abilities of male *Drosophila melanogaster* subjected to ELF–EMF exposure. Mutat Res Genet Toxicol Environ Mutagen. 2013;758(1–2): 95–103. 10.1016/j.mrgentox.2013.10.004 24157427

[13] Valadez-Lira JA, Medina-Chavez NO, Orozco-Flores AA, Heredia-Rojas JA, Rodriguez-de la Fuente AO, Gomez-Flores R, et al Alterations of immune parameters on *Trichoplusia ni* (Lepidoptera: Noctuidae) larvae exposed to extremely low-frequency electromagnetic fields. Environ Entomol. 2017;46(2): 376–82. 10.1093/ee/nvx037 28334331

[14] Rogers LE, Warren JT, Hinds NR, Gano KA, Fitzner RE, Piepel GF. Environmental studies of a 1100-kV prototype transmission line: an annual report for the 1981 study period. Richland (WA): Battelle Pacific Northwest Laboratories; 1982.

[15] Wellenstein G. The influence of high-tension lines on honeybee colonies (translation from the original German). J Appl Entomol. 1973;74: 86–94.

[16] Morse RA, Hooper T. The Illustrated Encyclopedia of Beekeeping. lst ed New York: Dutton Adult; 1985.

[17] Greenberg B, Bindokas VP, Frazier MJ, Gauger JR. Response of honey bees, *Apis mellifera* L., to high-voltage transmission lines. Environ Entomol. 1981;10(5): 600–10.

[18] Lee JM. Electrical and Biological Effects of Transmission Lines: A Review. Portland (OR): USDOE Bonneville Power Administration; 1989.

[19] Wright GA, Mustard JA, Simcock NK, Ross-Taylor AA, McNicholas LD, Popescu A, et al Parallel reinforcement pathways for conditioned food aversions in the honeybee. Curr Biol. 2010;20(24): 2234–40. 10.1016/j.cub.2010.11.040 21129969 PMC3011020

[20] Cappa F, Bruschini C, Protti I, Turillazzi S, Cervo R. Bee guards detect foreign foragers with cuticular chemical profiles altered by phoretic varroa mites. J Apic Res. 2016;55(3): 268–77.

[21] Goulson D, O’Connor ST, Park KJ. The impacts of predators and parasites on wild bumblebee colonies. Ecol Entomol. 2018;43(2): 168–81.

[22] Nouvian M, Reinhard J, Giurfa M. The defensive response of the honeybee *Apis mellifera*. J Exp Biol. 2016;219(22): 3505–17. 27852760 10.1242/jeb.143016

[23] Tan K, Dong S, Li X, Liu X, Wang C, Li J, et al Honey bee inhibitory signaling is tuned to threat severity and can act as a colony alarm signal. PLoS Biol. 2016;14(3): e1002423 10.1371/journal.pbio.1002423 27014876 PMC4807812

[24] Maschwitz UW. Alarm substances and alarm behaviour in social Hymenoptera. Nature. 1964;204(4956): 324.

[25] Collins AM, Rinderer TE, Tucker KW, Sylvester HA, Lackett JJ. A model of honeybee defensive behaviour. J Apic Res. 1980;19(4): 224–31.

[26] Núñez J, Maldonado H, Miralto A, Balderrama N. The stinging response of the honeybee: effects of morphine, naloxone and some opioid peptides. Pharmacol Biochem Behav. 1983;19(6): 921–4. 6657718 10.1016/0091-3057(83)90391-x

[27] Vergoz V, Roussel E, Sandoz JC, Giurfa M. Aversive learning in honeybees revealed by the olfactory conditioning of the sting extension reflex. PLoS One. 2007;2(3): e288 10.1371/journal.pone.0000288 17372627 PMC1810431

[28] McNally GP, Westbrook RF. Predicting danger: the nature, consequences, and neural mechanisms of predictive fear learning. Learn Mem. 2006;13(3): 245–53. 10.1101/lm.196606 16741278 PMC10807866

[29] Zhang E, Nieh JC. The neonicotinoid imidacloprid impairs honey bee aversive learning of simulated predation. J Exp Biol. 2015;218(20): 3199–205. 26347552 10.1242/jeb.127472

[30] Breed MD. Nestmate recognition in honey bees. Anim Behav. 1983;31(1): 86–91. 10.1006/anbe.1997.0581 9480667

[31] Richard FJ, Holt HL, Grozinger CM. Effects of immunostimulation on social behavior, chemical communication and genome-wide gene expression in honey bee workers (*Apis mellifera*). BMC Genomics. 2012;13(1): 558. 23072398 10.1186/1471-2164-13-558 PMC3483235

[32] Li-Byarlay H, Rittschof CC, Massey JH, Pittendrigh BR, Robinson GE. Socially responsive effects of brain oxidative metabolism on aggression. Proc Natl Acad Sci USA. 2014;111(34): 12533–7. 10.1073/pnas.1412306111 25092297 PMC4151721

[33] Rittschof CC, Coombs CB, Frazier M, Grozinger CM, Robinson GE. Early-life experience affects honey bee aggression and resilience to immune challenge. Sci Rep. 2015;5: 15572 10.1038/srep15572 26493190 PMC4616062

[34] Snodgrass RE. Anatomy and physiology of the honey bee. London: Constable and Company; 1956.

[35] Dade HA. Anatomy and dissection of the honeybee. Cardiff: International Bee Research Association; 1962.

[36] Ogawa H, Kawakami Z, Yamaguchi T. Motor pattern of the stinging response in the honeybee *Apis mellifera*. J Exp Biol. 1995;198(1): 39–47. 9317296 10.1242/jeb.198.1.39

[37] Menzel R, Müller U. Learning and memory in honeybees: from behavior to neural substrates. Annual Rev Neurosci. 1996;19(1): 379–404. 8833448 10.1146/annurev.ne.19.030196.002115

[38] Hammer M. The neural basis of associative reward learning in honeybees. Trends Neurosci. 1997;20(6): 245–52. 10.1016/s0166-2236(96)01019-3 9185305

[39] Hammer M, Menzel R. Multiple sites of associative odor learning as revealed by local brain microinjections of octopamine in honeybees. Learn Mem. 1998;5(1): 146–56. 10454379 PMC311245

[40] Jarriault D, Fuller J, Hyland BI, Mercer AR. Dopamine release in mushroom bodies of the honey bee (*Apis mellifera* L.) in response to aversive stimulation. Sci Rep. 2018;8(1): 16277 10.1038/s41598-018-34460-1 30389979 PMC6214997

[41] Nouvian M, Mandal S, Jamme C, Claudianos C, d’Ettorre P, Reinhard J, et al Cooperative defence operates by social modulation of biogenic amine levels in the honey bee brain. Proc Biol Sci. 2018;285(1871): 20172653 10.1098/rspb.2017.2653 29367399 PMC5805953

[42] Todorović D, Marković T, Prolić Z, Mihajlović S, Rauš S, Nikolić L, et al The influence of static magnetic field (50 mT) on development and motor behaviour of Tenebrio (Insecta, Coleoptera). Int J Radiat Biol. 2013;89(1): 44–50. 10.3109/09553002.2012.715786 22849716

[43] Jankowska M, Pawlowska-Mainville A, Stankiewicz M, Rogalska J, Wyszkowska J. Exposure to 50 Hz electromagnetic field changes the efficiency of the scorpion alpha toxin. J Venom Anim Toxins Incl Trop Dis. 2015;21(1): 38. 26430395 10.1186/s40409-015-0040-9 PMC4589959

[44] Hermann HR. Sting autotomy, a defensive mechanism in certain social Hymenoptera. Insectes Soc. 1971;18(2): 111–20.

[45] Cunard SJ, Breed MD. Post-stinging behavior of worker honey bees (Hymenoptera: Apidae). Ann Entomol Soc Am. 1998;91(5): 754–7.

[46] Russell KN, Ikerd H, Droege S. The potential conservation value of unmowed powerline strips for native bees. Biol Conserv. 2005;124(1):133–48.

[47] Wojcik VA, Buchmann S. Pollinator conservation and management on electrical transmission and roadside rights-of-way: a review. J Pollinat Ecol. 2012;7.

[48] Wagner DL, Ascher JS, Bricker NK. A transmission right-of-way as habitat for wild bees (Hymenoptera: Apoidea: Anthophila) in Connecticut. Ann Entomol Soc Am. 2014;107(6): 1110–20.

[49] Berg Å, Bergman KO, Wissman J, Żmihorski M, Öckinger E. Power-line corridors as source habitat for butterflies in forest landscapes. Biol Conserv. 2016;201: 320–6.

[50] Hill B, Bartomeus I. The potential of electricity transmission corridors in forested areas as bumblebee habitat. R Soc Open Sci. 2016;3(11): 160525 10.1098/rsos.160525 28018640 PMC5180138

