# Supplementary material for: Increased aggression and reduced aversive learning in honey bees exposed to extremely low frequency electromagnetic fields
Source: PLoS One. 2019 Oct 10;14(10):e0223614. doi: 10.1371/journal.pone.0223614 (PMC6786539; doi:10.1371/journal.pone.0223614)
Supplement: S1 Table — (DOCX) [file pone.0223614.s001.docx]

**S1 Table.** **The number of bees in SER analyses (after exclusions) for each hive and treatment**

| **Treatment** | **Hive** | **Bees/Hive** | **Bees/Treatment** |
| --- | --- | --- | --- |
| Control | 1 | 39 | 113 |
|  | 2 | 37 |  |
|  | 3 | 37 |  |
| 100µT | 1 | 39 | 114 |
|  | 2 | 38 |  |
|  | 3 | 37 |  |
| 1000µT | 1 | 38 | 114 |
|  | 2 | 36 |  |
|  | 3 | 40 |  |
