# Supplementary material for: Increased aggression and reduced aversive learning in honey bees exposed to extremely low frequency electromagnetic fields
Source: PLoS One. 2019 Oct 10;14(10):e0223614. doi: 10.1371/journal.pone.0223614 (PMC6786539; doi:10.1371/journal.pone.0223614)
Supplement: S2 Table — (DOCX) [file pone.0223614.s002.docx]

**S2 Table. The number of bees in intruder assay analyses for each hive and treatment**

| **Treatment** | **Hive** | **Replicates/Hive** | **Bees/Replicate** | **Bees Assayed** |
| --- | --- | --- | --- | --- |
| Control | 1 | 6 | 10 | 60 |
|  | 2 | 6 | 10 | 60 |
|  | 3 | 6 | 10 | 60 |
|  | 4 | 6 | 10 | 60 |
|  | 5 | 6 | 10 | 60 |
| 100µT | 1 | 6 | 10 | 60 |
|  | 2 | 6 | 10 | 60 |
|  | 3 | 6 | 10 | 60 |
|  | 4 | 6 | 10 | 60 |
|  | 5 | 6 | 10 | 60 |
